# Imaging technologies for the detection of sinus pathologies of odontogenic origin. A review

**DOI:** 10.21142/2523-2754-0901-2021-049

**Published:** 2021-03-11

**Authors:** Óscar Lozano González, Marco Felipe Salas Orozco

**Affiliations:** 1 Division of Oral and Maxillofacial Radiology, Universidad Científica del Sur. Lima, Perú. oscarlozanogonzalez@gmail.com Universidad Científica del Sur Division of Oral and Maxillofacial Radiology Universidad Científica del Sur Lima Peru oscarlozanogonzalez@gmail.com; 2 Division of Orthodontics, School of Dentistry, Universidad Autónoma de San Luis Potosí. San Luis Potosí, México. marco-salas@hotmail.com Universidad Autónoma de San Luís Potosí Division of Orthodontics, School of Dentistry Universidad Autónoma de San Luis Potosí San Luis Potosí Mexico marco-salas@hotmail.com

**Keywords:** imaging, sinus pathologies of odontogenic origin, conebeam computed tomography, review, imagen, patologías sinusales de origen odontogénico, tomografía computarizada de haz cónico, revisión

## Abstract

Sinus pathologies of odontogenic origin (SPO) are common in the clinical consultation; however, the dentist has some complications to detect them because their discovery is usually incidental and through imaging studies that, in most cases, are of low quality. The objective of this review is to describe the pertinent imaging resources that allow the detection of the most frequent SPO and, at the same time, carry out an updated review of the scientific literature in order to recognize the imaging of both the maxillary sinus and the dental organs. The scientific literature focused on this topic, published between 2014 and 2020, was consulted. The review showed two important results: the first is that Cone Beam Tomography (CBCT) represents the imaging modality with the best performance for the detection of SPO by what can be considered the gold standard for this purpose. The second is that the most frequent SPO is sinus mucositis, which is related to odontogenic conditions such as periapical lesions and periodontal affectations. Although Cone Beam Computed Tomography (CBCT) is the most appropriate tool to detect SPO compared to images obtained by 2D devices, there are also other alternatives such as magnetic resonance imaging and ultrasonography, which seem to have a promising future.

## RESUMEN

Las patologías sinusales de origen odontogénico (PSO) son frecuentes en la consulta clínica; sin embargo, el odontólogo tiene algunas complicaciones para detectarlos porque su descubrimiento suele ser incidental y mediante estudios de imagen que, en la mayoría de los casos, son de baja calidad. El objetivo de esta revisión es describir los recursos de imagen pertinentes que permitan la detección de las PSO más frecuentes y, al mismo tiempo, realizar una revisión actualizada de la literatura científica con el fin de reconocer la imagenologia tanto del seno maxilar como de los órganos dentales. Se consultó la literatura científica centrada en este tema, publicada entre 2014 y 2020. La revisión arrojó dos resultados importantes: el primero es que la tomografía de haz cónico (TCHC) representa la modalidad de imagen con mejor desempeño para la detección de PSO, por lo que se puede considerar el estándar de oro para este propósito. La segunda es que la PSO más frecuente es la mucositis sinusal, que se relaciona con afecciones odontogénicas como lesiones periapicales y afectaciones periodontales. Si bien la TCHC es la herramienta más adecuada para detectar la SPO en comparación con las imágenes obtenidas con dispositivos 2D, también existen otras alternativas como la resonancia magnética y la ecografía, que parecen tener un futuro prometedor.

## INTRODUCTION

Sinus Pathologies of Odontogenic Origin (SPOO) can be defined as an inflammatory or infectious process that has its origin in the dental and dento alveolar structures and can directly or indirectly affect the Sinus Membrane (SM), which causes pathologies in the maxillary sinus ^1^. Frequently, dentists and otolaryngologists are unable to diagnose SPOO, and their identification is usually incidental ^2^. Only between 60 and 70% of cases are analyzed and diagnosed by medical radiologists, and the data have shown that the appearance of SPOO is mostly unilateral ^3^^-^^9^. Because there is a considerable variety of pathologies in the maxillary sinus, it has been reported that only between 10 and 12% of these pathologies have an odontogenic origin ^3^. The most frequent among are Schneiderian membrane mucositis, maxillary sinusitis, mucous retention cyst, and antral pseudocysts ^4^. The topographic relationship of the teeth concerning the maxillary sinus is not a determining factor for the appearance of pathologies, but they may be due to their anatomical relationship ^5^. For example, the average distance between the maxillary sinus and the dental apices is 1.97 mm ^3^. This can facilitate the spread of dental infections and iatrogenic acts (e. g. foreign body projection) ^(5, 10)^. The symptomology related to SPOO is variable ^11^ and only 29% of the patient’s present pain. Establishing a strictly clinical diagnosis is complicated ^12^ and there is a risk of subjecting the patient to unnecessary pharmacological regimens and surgical procedures ^1^. Periapical and panoramic radiographs have been the most widely used imaging tools to diagnose SPOO. However, both have a low predictive value due to their two-dimensional nature and high distortion ^11^. For this reason, currently, the Cone Beam Computed Tomography (CBCT) has proven to be the most appropriate tool for the identification and diagnosis of SPOO, due to the high resolution of its images and the considerably lower radiation compared to a medical tomography ^1^^,^^3^^,^^5^^,^^13^^,^^14^. In addition, CBCT can show the exact size and location of the pathology, its relationship with the anatomical structures, and establish treatment plans. Thus, the purpose of this review is to describe which are the most effective and convenient imaging tools for the detection of sinus pathologies of odontogenic origin.

## ARTICLE SELECTION

The bibliographic research was carried out in databases such as Medline (PubMed), Scopus, LILACS, SciELO, Google Scholar and Cochrane Library. The studies published between 2014 and 2020 were consulted, the search terms used were: "sinusitis of the maxillary sinus and dental organs", "sinusitis of odontogenic origin", "sinus pathologies of odontogenic origin", "periodontitis and maxillary sinusitis", "apical periodontitis and maxillary sinusitis" , “unilateral sinusitis of odontogenic origin” and “odontogenic conditions and pathologies of the maxillary sinus”. In addition, both prospective and retrospective reviews and studies were consulted.

## IMAGING OF THE MAXILLARY SINUS AND ITS RELATIONSHIP WITH TEETH

The anatomical structures present between the maxillary sinus and the dental organs are the apices of the dental roots, the periodontium, the alveolar bone, and the vascular canals. Due to the relationship between the airways and the dental organs, the maxillary sinus should be inspected by both the otolaryngologist and the dental surgeon ^15^^,^^16^. Th e Sinus Membrane (SM) is found inside the maxillary sinus. It has a thickness of 0.3 to 0.8 mm, its nature is similar to the nasal mucosa, and it is composed of the pseudostratified epithelium with ciliary cells. This membrane can suffer morphological alterations due to an infectious process or be perforated during a surgical procedure. Therefore, its study has become clinically important ^17^^,^^18^. The maxillary sinus is an anatomical structure; its size and shape vary according to age, height, and pneumatization degree ^6^^,^^19^. It is closely related to the apices of the posterior maxillary dental organs. It is essential to take this into account because-on some occasions- it can generate a direct communication between the septic environment of an infected dental organ and the sinus cavity and favor the appearance of an acute or chronic inflammatory process. Concerning the maxillary sinus, the first premolar of the posterior teeth is the furthest away, and the mesiobuccal root of the upper second molar, the closest ^20^. Different proposals have been made to classify the proximity between the root apices and the maxillary sinus floor. One of them, is the one developed by Jung in 2009, which proposes four different classifications based on the use of the CBCT. Type 0: the floor of the maxillary sinus is located on the root apex, type 1: the root apex touches the floor of the maxillary sinus, type 2: the floor of the maxillary sinus is interposed between the roots, and type 3: apical protrusion over the maxillary sinus. ^7^^,^^19^ The relationship between the upper posterior dental organs’ root apices and the maxillary sinus has been reported with a frequency between 10.5 and 34.2%. In 46% of incidence cases, a part of the root extends beyond the maxillary sinus, being the most frequent roots the palatal root of the upper first molar and the mesiobuccal root of the upper second molar ^21^. The canal for the intraosseous branch of the Posterior Superior Alveolar Artery (rioAASP) is one of the most important anatomical landmarks at the level of the dental organs and the maxillary sinus because it is extremely crucial during the execution of surgical procedures like implant placement, maxillary sinus floor elevation, or periapical surgery. The presence of this artery has been reported employing the CBCT in 56.2% of the patients. In 32% of these, the artery appears to have a diameter greater than 1mm ^22^. Via the CBCT, it has been revealed that the rioPSAA travels through an intraosseous canal with an average distance of 6.18 mm from the dental apices and 6.41mm from the maxillary sinus floor. This information is critical to avoid the risk of laceration during a surgical procedure ^20^. Similarly, the CBCT has revealed that the volume of the maxillary sinus is totally or partially decreased in edentulous patients. This decrease may be due to the fact that the roots of the dental organs function as a natural support for the anteroposterior architecture of the maxillary bone; therefore, if they are absent, it results in a decreased sinus volume ^23^. Apparently, other factors that influence the volume of the maxillary sinus are gender and age. Gender, since some reports indicate that it is significantly higher in men than in women. And age, since sinus volume is lower in patients older than 24 years due to the fact that the full growth of the maxillary sinus occurs between the second and third decade of life ^24^. A recent study carried out with CBCT reported that there is no significant difference regarding the presence of sinus septa in totally or partially edentulous patients. However, it was found that the majority of septa are located in the region posterior to the level of the second upper molar. This information is very relevant and vital to consider for clinical because it prevents operative complications during implant placement; therefore, it could be said that the implemented CBCT is helpful to establish an adequate treatment plan ^25^. Finally, it has been shown that via the use of the CBCT, even though it is commonly spoken of pneumatization of the maxillary sinus after performing a dental extraction, the changes in the maxillary sinus floor position are minimal. Furthermore, most of the structural alterations occur at the bone crest level, which would have a direct clinical implication that would opt for a therapeutic option that minimizes this remodeling, such as alveolar preservation ^26^.

## IMAGING ASPECTS OF SINUS PATHOLOGIES OF ODONTOGENIC ORIGIN

Between 10 and 12% of all cases of maxillary Sinusitis are of Odontogenic origin (SO) ^27^. During long term odontogenic infection, resorption of the alveolar bone may occur as a result of bacterial secretion of collagenase developed through the apical foramen, which can lead to direct communication between the dental organ and the sinus cavity ^(28, 29)^. Th rough CBCT, it has been possible to verify other factors such as frequency, location, and odontogenic conditions related to radio densities that were previously detected incidentally. Using CBCT, SM has been evaluated to detect the presence of periapical lesions in upper maxillary teeth. The results show that the most common type of thickening in the floor of the maxillary sinus is of the “flat” type; and the related odontogenic conditions were mostly periodontal disease and periapical pathologies; ^30^ and it is usually more frequent in the upper first molar (44% of cases), and in the upper second molars (33%), which is due to the fact that both dental organs have a close relationship with the maxillary sinus ^31^. Thus, CBCT has proven to be a useful tool to determine the prevalence of SPO in cases of unilateral sinusitis; for example, in one study, 45% of SPO of the total SU was found, which, in addition, were related to pathologies in the ethmoid and frontal sinuses ^9^. Th e CBCT has made it possible to assess the structural changes in SM cases after one year of having performed root canal treatment in infected dental organs, so it is possible to visualize a statistically significant difference in which there is a 50% reduction in SM in most of the cases analyzed. For this reason, the authors suggest the selection of an adequate Field of View (FOV) to make a correct evaluation of this phenomenon; in this case, the small FOV would be ideal for evaluating a specific dental organ ^32^. Recently, CBCT was used as a tool to evaluate the relationship between the position of the maxillary sinus floor and the development of maxillary sinusitis in 152 patients. Based on these data, the horizontal relationship between it and the dental roots was classified into three divisions. Type 1: the maxillary sinus floor is positioned towards the vestibular area concerning the maxillary buccal roots. Type 2: the maxillary sinus is located on the vestibular root and the palatal. Type 3: the floor of the maxillary sinus is positioned towards the palatal concerning the palatal root. Because previous studies have frequently referred to the upper first molar as the tooth most commonly associated with odontogenic sinus infections, the authors took it as a reference for testing. However, it was shown that type 2 of the sinus floor’s horizontal configuration is the most related to inflammatory processes ^33^. Utilizing the CBCT, the odontogenic conditions that predispose SPO development have been analyzed, such as inadequate endodontic treatment, periapical lesion, and periodontal bone loss. They were classified as follows: localized or generalized Mucosal Thickening (SM), Maxillary Sinusitis (SM), and Mucosal Retention Cyst (MRN). From this analysis, it was concluded that the most prevalent SPO was generalized SD, which had an evident relationship with periodontal bone loss and the presence of periapical lesions ^5^.

## IMAGING TOOLS USED FOR THE DETECTION OF SINUS PATHOLOGIES

The importance of choosing high-precision imaging tools lies in need to make an adequate diagnosis and obtain predictable results when proposing a specific therapy. For this to be possible, it is essential to have early detection of SPO through radiological images. Among all the alternatives, the most used are; Panoramic Radiography (RP), periapical radiography, Cone Beam Computed Tomography (CBCT), and Magnetic Resonance Imaging (MRI) ^3^^,^^4^. Currently, among the options as mentioned earlier, Panoramic Radiography is usually the most used in dentistry due to its low cost, its easy access, and the little radiation it uses. However, it presents some disadvantages, such as the superposition of anatomical structures, a high degree of distortion ^35^, and it only records the information obtained within an established focal corridor. Generally, the maxillary sinuses’ anteroposterior dimension exceeds that of the alveolar ridge, so a large amount of information is not accurately detected ^36^. Furthermore, it has been reported that maxillary sinuses with a diameter of less than 3 mm are challenging to detect using this imaging technique ^37^. Constantine carried out a study in which 714 patients, where the efficacy of both RP and CBCT to detect pathologies of the maxillary sinus, was analyzed. The results showed that the sensitivity of RP was 36.7%, and its specificity was 88.1%, so the authors concluded that RP is not a viable alternative for the correct diagnosis of SPO, ^36^ information that is supported by other authors ^38^. With obtaining all these results, the CBCT has positioned itself as the most used tool for the diagnosis of pathologies related to the maxillary sinuses. Therefore, over the years, more and more research has been carried out on this topic ^12^^,^^39^. The research shows that applying the CBCT has as its top quality, multiplanar image management with different slice thicknesses, which solves the problem generated by the superposition of images present in the RP. However, due to the amount of radiation it emits, it is also essential to establish appropriate selection criteria for its use ^34^. During the interpretation process supported by the use of CBCT, the doctor and dentist can perform a detailed analysis of the sinus anatomy, bone patterns and their alterations, the presence of intrasinus calcifications, and the extent of sinus pathology. Employing this method has shown that more than 70% of patients diagnosed with dental infections in the upper jaw have morphological alterations in the maxillary sinus ^39^. Currently, several authors propose Magnetic Resonance Imaging as a recommended precision imaging alternative for the diagnosis and treatment plan of sinus pathologies since the anatomical characterization of soft tissue with a high degree contrast has been reported as one of its main virtues ^41^. The main difference between MRI and CBCT is in their way of obtaining the radiological image: The first works through signals emitted by the protons of the water within a magnetic field; and the second by X-ray attenuation patterns ^40^. One of the MRI modalities is the Diffusion Weighted Magnetic Resonance (DWI), which is in charge of quantifying the values obtained through an Apparent Diffusion Coefficient (ADC). Through the ADC, random molecular movement is expressed in detail from water in an intracellular medium to which magnetic energy is applied ^41^. In this case, the ADC is recorded using colors that vary from red to blue, where the first indicates the highest degree of diffusion of the water molecule and the second the lowest. Currently, on the one hand, there are no studies related to the diagnosis of SPO through the use of Ultrasound (US); however, its reliability in the diagnosis of acute and subacute maxillary sinusitis has been verified. On the other hand, some authors analyzed a total of 148 maxillary sinuses in 74 patients through a prospective cohort study in which they used endoscopy as a method to corroborate the diagnosis. It was concluded that ultrasound is a fast, effective, and easy to use tool to evaluate the maxillary sinus ^42^.

## CONCLUSIONS

SPOO are of multifactorial etiology, and, on occasions, their clinical manifestations are minimal. Dental imaging is a fundamental aid for the clinicians since it allows them to establish a correct diagnosis and propose an adequate therapy. 

Through various scientific studies, it has been shown that two-dimensional radiological images have considerably lower performance compared to CBCT. For this reason, it is essential to consider new alternatives for the diagnosis of SPOO, such as MRI and US, as both show a promising future in the treatment of these pathologies.
